# Multimodal AI-based risk stratification for distant metastasis in nasopharyngeal carcinoma

**DOI:** 10.1016/j.esmoop.2025.105809

**Published:** 2025-09-24

**Authors:** J. Zhou, M.S. Wibawa, R. Wang, Y. Deng, H. Huang, Z. Luo, Y. Xia, X. Guo, L.S. Young, K.-W. Lo, N. Rajpoot, X. Lv

**Affiliations:** 1 State Key Laboratory of Oncology in South China, Guangdong Key Laboratory of Nasopharyngeal Carcinoma Diagnosis and Therapy, Guangdong Provincial Clinical Research Center for Cancer, Sun Yat-sen University Cancer Center, Guangzhou, China; 2 Tissue Image Analytics Centre, Department of Computer Science, University of Warwick, Coventry, UK; 3 Warwick Medical School, University of Warwick, Coventry, UK; 4 Department of Anatomical and Cellular Pathology, The Chinese University of Hong Kong, Hong Kong, China

**Keywords:** nasopharyngeal carcinoma, artificial intelligence, deep learning, precision medicine

## Abstract

**Background:**

The TNM (tumour–node–metastasis) staging system is the primary tool for treatment decisions in nasopharyngeal carcinoma (NPC). However, therapeutic outcomes vary considerably between patients, and guidelines for the management of distant metastasis treatment remain limited. This study aimed to develop and validate a deep learning-based risk score to predict NPC survival.

**Methods:**

We developed a graph for nasopharyngeal carcinoma (GNPC) risk score, a multimodal deep-learning-based digital score incorporating signals from both haematoxylin and eosin-stained tissue slides and clinical information. Digitised images of NPC tissue slides were represented as graphs to capture spatial context and tumour heterogeneity. The proposed GNPC score was developed and validated on 1949 patients from two independent cohorts.

**Results:**

The GNPC score successfully stratified patients in both cohorts, achieving statistically significant results for distant metastasis (*P* < 0.001), overall survival (*P* < 0.01), and local recurrence (*P* < 0.05). Further downstream analyses of morphological characteristics, molecular features, and genomic profiles identified several factors associated with GNPC-score-based risk groups.

**Conclusion:**

The proposed digital score demonstrates robust predictive performance for distant metastasis, overall survival, and local recurrence in NPC. These findings highlight its potential to assist with personalised treatment strategies and improve clinical management for NPC.

## Highlights

• Developed GNPC, a multimodal AI model combining clinical and histopathological data to stratify distant metastasis risk in NPC.
• GNPC score was validated on 1949 patients from two independent cohorts, demonstrating robust predictive performance.
• Accurately stratified patients by risk of distant metastasis (*P* < 0.001), OS (*P* < 0.01), and local recurrence (*P* < 0.05).
• The study identified morphological, molecular, and genomic factors associated with GNPC-score-based risk groups.
• Potential to guide personalised treatment strategies and improve clinical management in nasopharyngeal carcinoma.

## Introduction

Nasopharyngeal carcinoma (NPC) is a malignant tumour of the head and neck that arises from the epithelium of the nasopharynx.^1^ Although it originates from similar tissue regions, NPC is distinctly different from other head and neck tumours. NPC mortality has decreased with the widespread use of radiotherapy and chemotherapy, and the 5-year survival has generally improved.^2^ The primary challenges in treatment and leading causes of mortality are local recurrence and distant metastasis. Furthermore, an additional 15%- 30% of those treated for locally advanced NPC will later develop local recurrence and/or distant metastasis.^3^

Treatment outcomes of NPC for the same treatment can vary significantly among patients with the same TNM (tumour–node–metastasis) stage.^4^^,^^5^ The variations in prognosis may be attributed to biological and tumour heterogeneity. Histological assessment remains the gold standard for cancer diagnosis and prognosis. Digital pathology has revolutionised this process by enabling histological tissues to be scanned into high-resolution images, known as whole slide images (WSIs). WSIs have been widely utilised for the application of artificial intelligence (AI) in computational pathology (CPath) for various tasks, including detection of tumour structure,^6^^,^^7^ cancer grading,^8^^,^^9^ molecular pathway prediction,^10^ and cancer prognosis.^11^

Compared with other types of cancer, CPath studies in NPC that utilise WSIs as a data modality are far less common. In diagnostic tasks, WSIs have been used to differentiate between benign and malignant NPC tumours using deep learning (DL) approaches.^12^ For prognostic tasks, microscopic pathological features extracted from WSIs have been employed to generate risk scores for progression-free survival in NPC patients.^13^ Another study integrated clinical factors, histopathological features, and radiomic signatures to develop a multiscale nomogram for predicting failure-free survival.^14^ Additionally, DL has been applied for the automated quantification of tumour-infiltrating lymphocytes (TILs) to stratify patient risk effectively.^15^ However, the generalisability of these studies is yet to be validated due to the small or single-centric cohorts.

This study aims to develop a novel biomarker for NPC by leveraging DL to integrate clinical information and histological images within a unified framework. We propose a graph neural network model for NPC (GNPC) risk score that takes graph representations of WSIs and combines features from a foundation model with morphological characteristics to represent the tumour microenvironments (TMEs). Additionally, molecular and genomic data were utilised to further investigate the results of GNPC-score-based risk stratification, analysing the association of specific genetic mutations with immune cell infiltration and tumour nuclear morphology. We employed two independent cohorts to train and evaluate our model. To the best of our knowledge, this is the first multicentric study to apply DL with WSIs for patient prognostication in NPC.

## Materials and methods

### Data

We retrospectively collected WSIs and clinical data from two cohorts: the Sun Yat-sen University Cancer Centre (SYSUCC; *n* = 2072 patients) and the Chinese University of Hong Kong (CUHK; *n* = 145 patients), with a total of 2428 WSIs. All NPC tumours from the CUHK cohort were collected by endoscopy or surgery at the CUHK, Hong Kong, SAR. Following a data cleaning process, the final cohort included 1849 patients from SYSUCC and 100 patients from CUHK. Detailed information on the clinicopathological information (Supplementary Table S1, available at https://doi.org/10.1016/j.esmoop.2025.105809) and data exclusion process (Supplementary Figure S1, available at https://doi.org/10.1016/j.esmoop.2025.105809) is available in the Supplementary Material, accesible at https://doi.org/10.1016/j.esmoop.2025.105809. Molecular data such as Epstein–Barr virus (EBV) DNA data were available only in the SYSUCC cohort, whereas latent membrane protein 1 (LMP1) status and genomic data were available in the CUHK cohort.

### Graph for nasopharyngeal carcinoma risk stratification (GNPC)

**Figure 1:**
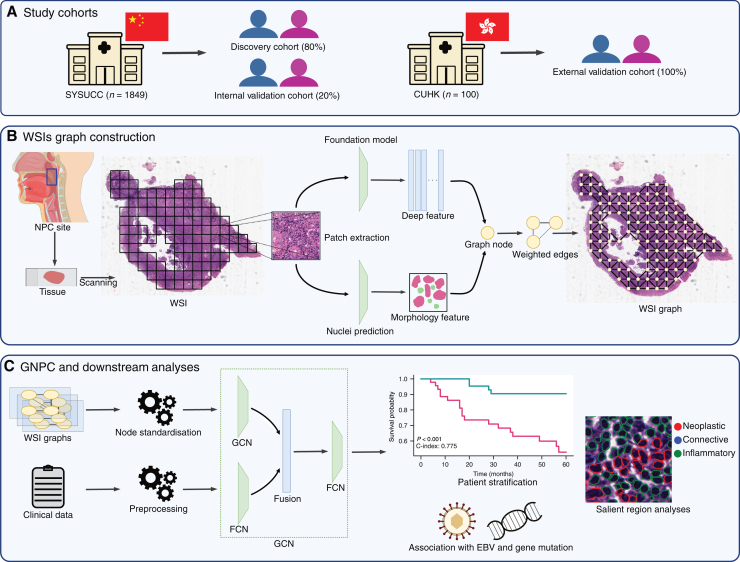
**Main pipeline of the study.** (A) Two cohorts were utilised as one discovery and two validations. (B) Graphs were constructed from node of deep features and nuclei morphology features. (C) Multimodal model learns from whole slide image (WSI) graphs and clinical data to generate risk score of nasopharyngeal cancer (NPC). CUHK, Chinese University of Hong Kong; EBV, Epstein–Barr virus; FCN, fully connected layer; GCN, graph convolutional network; GNPC, graph for nasopharyngeal carcinoma; SYSUCC, Sun Yat-sen University Cancer Centre.

From each WSI, we extracted image patches from tissue regions at a resolution of 0.5 μm per pixel (mpp). Leveraging the power of a foundation model, we extracted deep features from these image patches, enabling a rich representation of the underlying pathology. Based on our comparative analysis (Supplementary Figure S3, available at https://doi.org/10.1016/j.esmoop.2025.105809), we chose CONCH^16^ as the feature extractor. Additionally, nuclei within each patch were detected and classified. As shown in Figure 1, we constructed graphs from nodes that combine deep features and morphological features of tumour and inflammatory nuclei.

GNPC is a multimodal model wherein the latent embeddings of tissue patches and clinical data are amalgamated to generate a risk score (Supplementary Figure S2, available at https://doi.org/10.1016/j.esmoop.2025.105809). The latent embeddings from the graph convolutional network (GCN) and fully connected layer (FCN) were concatenated and passed into the FCN to generate risk scores, with a higher score corresponding to a higher risk of the event.

### Model evaluation

The primary endpoint was distant-metastasis-free survival (DMFS), calculated from the date of diagnosis to the date of the event. Additional endpoints included overall survival (OS) and local-recurrence-free survival (LRFS). Survival data from both cohorts were right-censored, with a maximum observed follow-up period of 5 years.

Patients from the SYSUCC cohort were divided into a discovery set (80%) and an internal validation set (20%). All patients in the CUHK cohort were used as the external validation set. The GNPC model was trained on the discovery set and evaluated on both the internal and external validation sets (Supplementary Figure S4, available at https://doi.org/10.1016/j.esmoop.2025.105809). Hyperparameters of the models were optimised using cross-validation within the discovery set. Patients in the internal and external validation cohorts were stratified into low- and high-risk groups based on the median risk score of GNPC derived from the discovery cohort. Patients with risk scores above the cut-off were classified as high risk, and vice versa.

### Ethics

Informed consent was obtained from all CUHK participants in accordance with institutional clinical research approvals. Data from SYSUCC and all procedures were conducted in accordance with ethical principles (B2023-381-01).

## Results

### Prognostic value of GNPC

**Figure 2:**
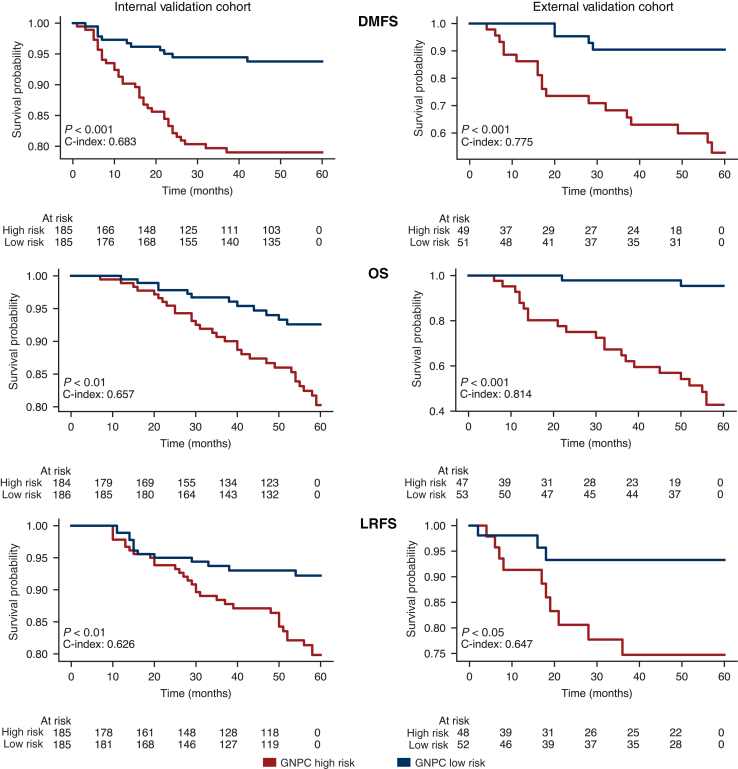
**Survival prediction with graph for nasopharyngeal carcinoma (GNPC).** GNPC evaluation with Kaplan–Meier curves on two validation cohorts for distant-metastasis-free survival (DMFS), overall survival (OS), and local-recurrence-free survival (LRFS) cases. Significance levels denoted as *P* < 0.05, *P* < 0.01, and *P* < 0.001.

As shown in Figure 2, for 5-year DMFS, our model achieved a C-index of 0.683 and 0.775 in the internal and external validation cohorts, respectively. Additionally, a log-rank test yielded *P* < 0.001 for both cohorts, indicating that the GNPC has statistically significant prognostic power in patient risk stratification. For 5-year OS, the GNPC score achieved C-indices of 0.657 and 0.814 in the internal and external validation cohorts, respectively. The log-rank test indicated statistical significance, with *P* < 0.01 in the internal cohort and *P* < 0.001 in the external cohort. Furthermore, for 5-year LRFS, our model achieved a C-index of 0.626 in the internal validation cohort (*P* < 0.01) and 0.647 in the external cohort (*P* < 0.05). We also found that our risk score consistently outperformed TNM staging, achieving the highest C-index across all survival endpoints in both validation cohorts (Supplementary Tables S2–S7, available at https://doi.org/10.1016/j.esmoop.2025.105809).

### Risk factors in NPC survival

**Figure 3:**
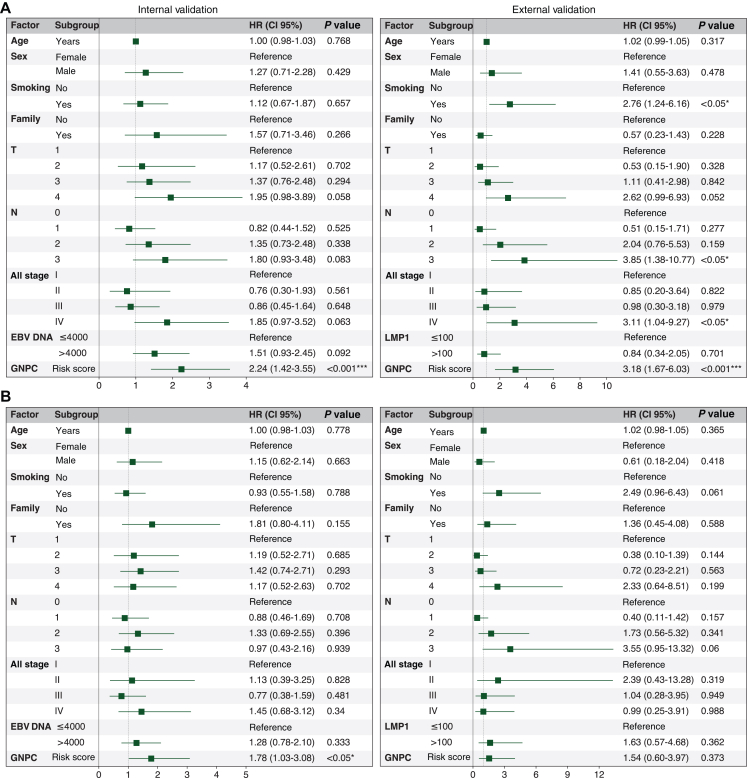
**Cox proportional hazards analysis in distant metastasis for both validation cohorts.** (A) Univariate and (B) multivariate. Significance levels denoted as ∗*P* < 0.05, ∗∗*P* < 0.01, and ∗∗∗*P* < 0.001. EBV, Epstein–Barr virus; GNPC, graph of nasopharyngeal cancer; LMP1, latent membrane protein 1; N, node; T tumour.

Univariate Cox proportional hazards regression analysis showed that the GNPC score in both the internal validation cohort (*P* < 0.001) and the external validation cohort (*P* < 0.001) was significantly associated with poor prognosis in distant metastasis cases (Figure 3). None of the other clinical factors were associated with distant metastasis prognosis in the internal validation cohort. Similar findings were observed for 5-year OS (Supplementary Figure S6, available at https://doi.org/10.1016/j.esmoop.2025.105809), where the GNPC score was significantly associated with poor prognosis in the internal validation cohort (*P* < 0.01) and the external validation cohort (*P* < 0.001). However, for cases of local recurrence (Supplementary Figure S7, available at https://doi.org/10.1016/j.esmoop.2025.105809), the GNPC score was significantly associated with poor prognosis only in the larger cohort (*P* < 0.01), with no significant association found in the external validation cohort (*P* = 0.125).

In multivariate Cox proportional hazards regression analysis, the GNPC score was found to be an independent risk factor in the internal validation cohort for DMFS (*P* < 0.001), OS (*P* < 0.05), and LRFS (*P* < 0.05), but not in the external validation cohort for DMFS (*P* = 0.373), OS (*P* = 0.197), or LRFS (*P* = 0.464). This discrepancy may be due to the limited sample size in the CUHK cohort.

### Association with risk factors and genomic alteration

Recent genomic studies indicate that NPC pathogenesis is characterised by frequent nuclear factor kappa-light-chain-enhancer of activated B cells (NF-*κ*B) activation and immune evasion in 90% of cases. Investigating how specific gene alterations impact NPC prognosis could reveal new therapeutic opportunities for this disease.

**Figure 4:**
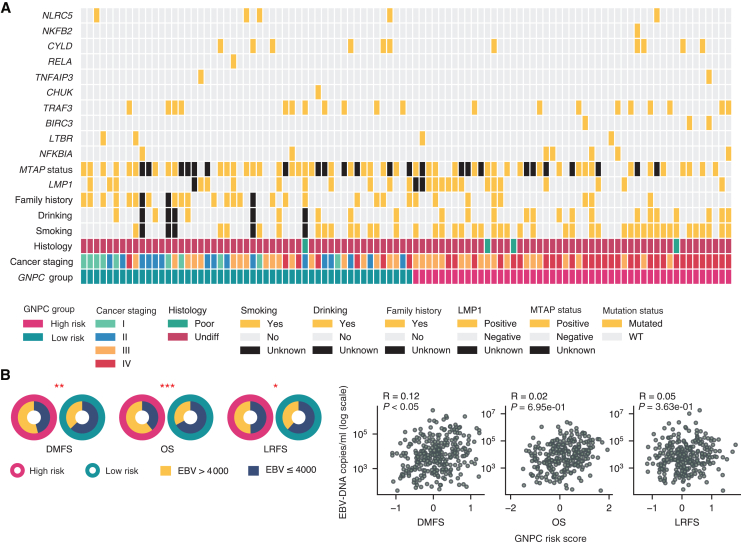
**GNPCassociation with NPC risk factors in distant metastasis cases.** (A) Heatmap of risk factors and graph of nasopharyngeal cancer (GNPC) risk groups in the external validation cohort. (B) Distribution comparison (left) and correlation between Epstein–Barr (EBV) DNA copies/group and GNPC risk groups/scores in the internal validation cohort. Statistical significance denoted by red star. Significance levels denoted as ∗*P* < 0.05, ∗∗*P* < 0.01, and ∗∗∗*P* < 0.001. DMFS, distant-metastasis-free survival; LMP1, latent membrane protein 1; LRFS, local-recurrence-free survival; OS, overall survival; WT, wild type.

We examined the distribution of genomic alterations within risk groups of GNPC across survival endpoints (Figure 4A, Supplementary Figures S8 and S9, available at https://doi.org/10.1016/j.esmoop.2025.105809), aiming to uncover molecular distinctions that may differentiate these groups. Although we found no statistically significant results, several genes showed consistent alteration patterns linked to risk group. For instance, alterations to *NFKBIA* and *BIRC3* were more prevalent in the high-risk GNPC group, while *NLRC5* alterations were more common in the low-risk group.

### GNPC risk score associated with EBV infection

We consistently found a higher number of cases with elevated EBV DNA in the high-risk group across all survival endpoints, with statistically significant results (*P* < 0.05), as illustrated in Figure 4B. This suggests that patients in the high-risk GNPC category are more likely to have elevated EBV DNA levels, which may indicate a link between EBV viral load and increased risk as assessed by the GNPC model. However, when directly correlating GNPC scores with EBV DNA levels, we observed only a weak but statistically significant relationship (R = 0.12, *P* < 0.05) specifically in DMFS. Nevertheless, the statistical significance in DMFS highlights a possible subtle association between EBV DNA levels and the likelihood of distant metastasis, although the effect size is limited.

EBV infection in NPC encodes several viral oncogenes, including LMP1. LMP1 exhibits immunomodulatory properties, involved in activating signalling pathways, such as NF-κB, that drive tumour growth and progression. We found no significant association between LMP1 expression levels and GNPC risk groups across all survival endpoints (Supplementary Figure S5, available at https://doi.org/10.1016/j.esmoop.2025.105809). This finding suggests that while LMP1 contributes to early NPC development, its expression does not appear to influence GNPC-based risk stratification outcomes.

### Salient region analyses

**Figure 5:**
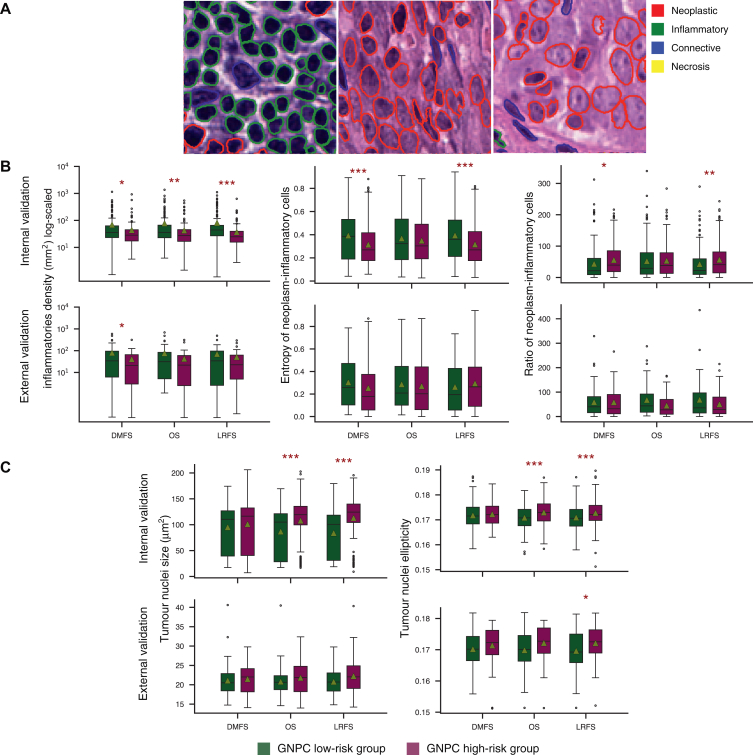
**Salient regions in whole slide images (WSIs) from top 10% patches.** (A) Examples of nuclei composition in relevant image patches contributing to survival prediction. (B) Immune profile of graph of nasopharyngeal cancer (GNPC) risk groups across all survival endpoints. (C) Tumour nuclear pleomorphism across GNPC risk groups. Statistical significance is denoted by a red star. Significance levels denoted as ∗*P* < 0.05), ∗∗*P* < 0.01, and ∗∗∗*P* < 0.001. DMFS, distant-metastasis-free survival; LRFS, local-recurrence-free survival; OS, overall survival.

The attention mechanism in the aggregation layer was used to select the top 10% most relevant patches for model prediction. As shown in Figure 5A, the top patches consisted of a high density of tumour and inflammatory cells, which is relevant to cancer progression.

### Higher density of immune cells in the lower risk group

Our findings (Figure 5B) indicate that lower risk groups tended to exhibit a higher average density of inflammatory cell infiltration across all survival endpoints in the validation cohort. This association was statistically significant across all survival endpoints in the internal validation cohort. However, in the external validation cohort, which had fewer cases, statistical significance was observed only for distant metastasis. Our model effectively captured the complex interplay between TILs and NPC progression, aligning with findings from previous studies.^17^

### Increased nuclear pleomorphism in the high-risk group

It has been suggested that neoplastic spindle cells, prominently located at the invasive tumour front and within the surrounding stroma, are associated with poor prognosis in NPC.^18^ Using the same pipeline as our analysis on inflammatory cell infiltration, we measured tumour ellipticity at the patient level (Figure 5C). We found that high-risk patients in the internal validation cohort showed larger nuclei with greater ellipticity in LRFS and OS cases with statistically significant result (*P* < 0.05).

### Association between immune characteristics and gene mutation in NPC

Recent studies highlight that specific gene mutations can significantly affect immune cell dynamics within tumours,^19^^,^^20^ potentially altering lymphocyte count. To further investigate this relationship, we analysed lymphocyte/inflammatory cell density across tumour regions with different gene mutation profiles in the CUHK cohort (Supplementary Figure S10, available at https://doi.org/10.1016/j.esmoop.2025.105809).

Across all survival endpoints, we observed no significant difference in inflammatory cell density between groups, except for lymphotoxin-beta receptor (*LTBR*). Patients with an *LTBR* amplification exhibited a higher inflammatory cell density, with statistically significant results. *LTBR* is crucial in both the development and regulation of the immune system.^21^ Previous studies have demonstrated *LTBR* as a driver of nonconical NF-κB activation in NPC.^19^^,^^22^ The association between *LTBR* amplification and increased inflammatory cells suggests a potential link between *LTBR*-mediated nonconical NF-κB activation and immune cell recruitment within the TMEs.

### Specific gene mutation associated with spindle tumour cells

Gene mutations may drive cancer progression, often leading to observable changes in tumour cell morphology that may reflect underlying biological processes, specifically, the NF-*κ*B pathway, which represents the transcription factor involved in the regularisation of tumour proliferation and apoptosis.^20^ By comparing morphological traits across mutation profiles, our analysis reveals a distinctive pattern in tumour nuclei shape with genetic alterations to *BIRC3* (Supplementary Figure S10, available at https://doi.org/10.1016/j.esmoop.2025.105809). The proportion of tumour nuclei exhibiting a spindle shape is significantly higher (*P* < 0.05) in the group of patients with altered *BIRC3* in the external validation cohort across all survival endpoints.

## Discussion

In this multicohort retrospective study, we introduce the digital GNPC risk score, which is based on multimodal graph learning for predicting distant metastasis risk in NPC. The GNPC risk score encodes complex interactions within the TME across slides and tissue areas into a unified graph by integrating foundation model features, cellular morphology, spatial location, and clinical information. The results demonstrate strong performance and generalisability in both validation cohorts for predicting distant metastasis in NPC. Additionally, it also achieved significant results in risk stratification for OS and local recurrence. These findings support the potential clinical utility of the score in accurately identifying high-risk patients who may benefit from closer monitoring or more aggressive therapeutic strategies, thereby providing valuable insights for individualised risk assessment and treatment planning.

Persistent EBV infection contributes to the progression of NPC by expressing viral proteins such as EBNA1 and the LMP proteins, which drives the oncogenic process, as well as disrupts immune surveillance mechanisms.^23^^,^^24^ NPC is characterised by significant TMEs consisting of stromal cells as a significant infiltration of immune cells. A higher infiltration of immune cells within the tumour area is associated with a better prognosis in NPC.^25^ In our analysis of salient regions of NPC, we discovered that the low-risk group demonstrates a higher level of immune cell activity compared with the higher risk GNPC groups. This finding underscores the potential of GNPC as a biomarker for identifying immune-active NPC in EBV-infected cases, which may benefit from immune-targeted therapies. Despite the important role of LMP1 in immune regulation of NPC, we did not observe an association between LMP1 status and GNPC risk group. This may be due to the limited number of cases in the cohort with LMP1 data, and other somatic gene alterations contributing to immune regulation and inflammation in the tumours. The correlation of LMP1 and genetic alterations with the GNPC requires further investigation in a large cohort of NPC samples in future.

Despite the widespread use of the World Health Organization (WHO) subtype system for clinical classification of NPC, a growing number of studies suggest that the current WHO classification is inadequate for predicting chemotherapy and radiotherapy outcomes.^26^^,^^27^ Morphological characteristics of neoplastic cells have been proposed as a potential factor for improving NPC prognosis, with a higher proportion of neoplastic spindle cells linked to worse outcomes. Our analysis of salient regions revealed that tumour nuclei in the high-risk GNPC group have larger nuclei and exhibit a spindle shape compared with those in the low-risk group. These changes are believed to reflect increased cell plasticity associated with epithelial-to-mesenchymal transition and epigenetic alteration during cancer progression. This indicates that while our model incorporates the WHO classification in its multimodal branch, it also aligns with recent studies highlighting the prognostic value of morphology of neoplastic spindle cells.

The development of NPC is influenced by a combination of host genetics, environmental factors, and EBV infection. Although no significant differences were observed in the GNPC risk group between mutated and nonmutated genes, certain genes, such as *NFKBIA* and *BIRC3*, were more frequently altered in the high-risk GNPC group across all survival endpoints. Our TME profiling in salient regions revealed a significantly higher immune cell density in the altered *LTBR* group. Furthermore, tumours in the altered *BIRC3* group exhibited more spindle-shaped cells. These findings align with the role of *LTBR* activation and inactivation of *BIRC2*, *BIRC3*, and *TRAF3* in the activation of the noncanonical NF-kB pathway. Mutations in *NFKBIA* (IκBα) and *BIRC3* (cIAP2) are believed to exert a more potent effect on NF-κB signalling and inflammation compared with other negative regulators of the pathway, such as *TRAF3* and *CYLD*. This dysregulation may promote tumour survival and progression. In particular, the loss of *BIRC3*/cIAP2 function in NPC is hypothesised to drive activation of the noncanonical NF-κB pathway.^28^ Additionally, independent studies have identified loss-of-function mutations in NF-κB negative regulators, including *NFKBIA*, in NPC tumours.^29^ Collectively, these findings suggest that dysregulated NF-κB signalling, driven by genetic alterations, is a central mechanism in NPC pathogenesis.

One limitation of our study is that molecular data, such as EBV DNA, LMP1, and genomic alterations, were available only for a single cohort rather than across both cohorts. This limitation restricted our ability to generalise findings related to the molecular profiles of NPC. Additionally, the data in this study were acquired solely from cohorts in Asia, where NPC is highly prevalent. This geographic concentration may limit the generalisability of our findings to other populations, such as those in Europe or the United States, where NPC characteristics and risk factors may differ. Moreover, the relatively small number of cases in the external validation cohort may have contributed to the nonsignificant results. Further validation with diverse and larger cohorts would be beneficial to confirm the applicability of our model across various demographic and regional backgrounds.
