# Supplementary material for: Multimodal AI-based risk stratification for distant metastasis in nasopharyngeal carcinoma
Source: ESMO Open. 2025 Sep 24;10(10):105809. doi: 10.1016/j.esmoop.2025.105809 (PMC12495093; doi:10.1016/j.esmoop.2025.105809)
Supplement: Supplementary Material [file mmc1.pdf]

Data description

Cases from SYSUCC cohort were diagnosed and treated between January 2011 and December 2018 at Sun Yat-sen University Cancer Center (Guangzhou, China). The median follow-up duration in the SYSUCC cohort was 76 months (range: 1–135 months), while in the CUHK cohort, it was 55 months (range: 1–222 months). In both cohorts, male patients accounted for more than 70% of the cases.

Plasma EBV DNA levels were quantified using quantitative polymerase chain reaction targeting the BAMHI-W region of the EBV genome prior to treatment. Results were reported as the concentration of EBV genome copies per millilitre of plasma. Details regarding the collection of LMP1 status and genomic data can be found in these studies [1,2].

Table S1 Clinicopathological characteristics of cohorts

Covariates	Sub-covariates	SYSUCC*	CUHK*
Age		46.10 (10.97)	52.79 (12.47)
Sex	Female	497 (27%)	24 (24%)
	Male	1352 (73%)	76 (76%)
T	1	214 (12%)	24 (24%)
	2	240 (13%)	17 (17%)
	3	1039 (56%)	37 (37%)
	4	356 (19%)	22 (22%)
N	0	186 (10%)	18 (18%)
	1	714 (39%)	32 (32%)
	2	594 (32%)	33 (33%)
	3	355 (19%)	17 (17%)
All Stage	I	50 (3%)	10 (10%)
	II	205 (11%)	16 (16%)
	III	942 (51%)	37 (37%)
	IV	652 (35%)	37 (37%)
EBVDNA	≤ 4,000	1044 (56%)	-
	> 4,000	805 (44%)	-
LMP1	≤ 100	-	72 (74%)
	> 100	-	25 (26%)

* mean (std) for numerical data or number of count (percentage) for categorical data

Exclusion criteria

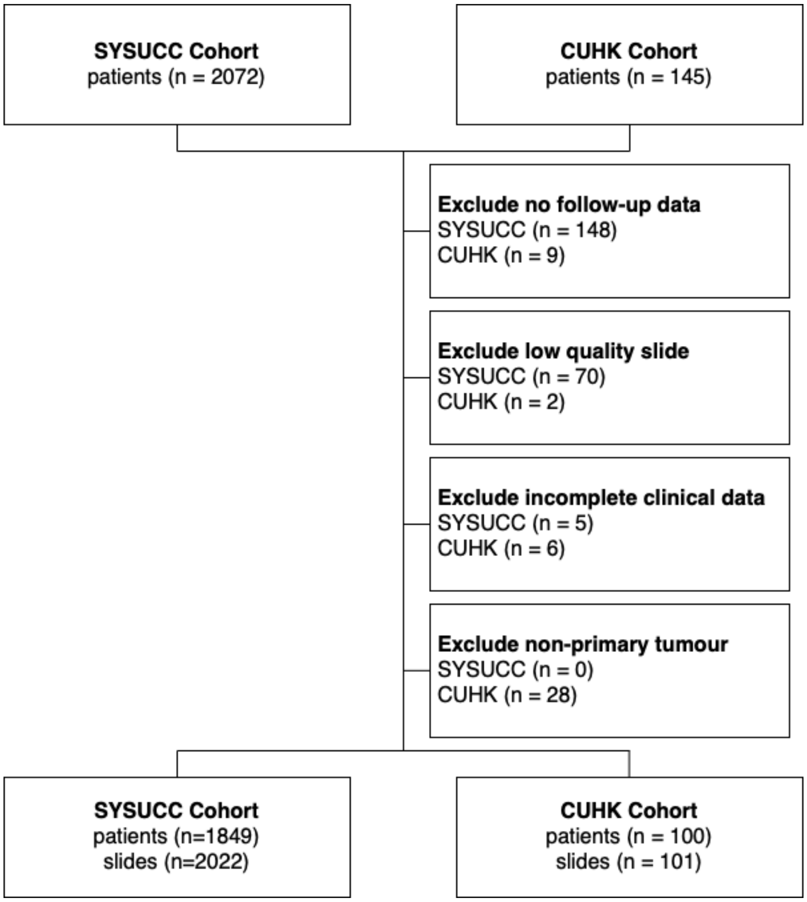

Figure S1 Consort diagram

GNPC Pipeline

Data preprocessing

To ensure the quality and integrity of the WSIs, a quantitative review was conducted on all WSIs using HistoQC [3]. WSIs that exhibited poor quality, such as those with significant blurriness, a high prevalence of image artifacts, or those containing only minimal tissue areas, were identified and excluded from the analysis. Patients without available follow-up data, those with incomplete clinical information (such as missing details regarding sex, cancer staging parameters [T, N, stage], or treatment history), and patients with non-primary tumours were excluded.

Image patches as graph nodes

Separation between tissue and non-tissue areas was performed at a lower magnification using Otsu thresholding for image segmentation. Following image segmentation, small objects and holes were removed using scikit-image to eliminate image artifacts and

improve quality.

Non-overlapping patches, each sized 256x256 pixels at 20x magnification or 0.5 mpp, were extracted from all tissue regions in the WSIs. These patches are the representation of nodes in the WSI graph. Node features, consisting of deep features and nuclei morphology were extracted in the subsequent data pipeline.

Deep feature extraction

Pathology images are inherently distinct from natural images, as they tend to exhibit low colour variation and lack canonical orientation. These characteristics can reduce the effectiveness of pretrained features, originally trained on natural images, when applied in the pathology domain. The advancement of self-supervised learning (SSL) enables the utilization of unlabelled data, which is particularly valuable in pathology, where annotations are costly to obtain. SSL can leverage unannotated pathology images to generate richer and more meaningful feature representations.

Owing to this, we employed four state-of-the-art SSL models, trained on histopathology data, to extract deep features from the patches. The SSL models used were VIRCHOW [4], CONCH [5], UNI [6] and DINO [7]. For comparison, we also used deep features extracted from the ResNet50 model, pretrained on ImageNet. The feature vector lengths for each model were: 512 for CONCH, 384 for DINO, 1024 for UNI, 2048 for ResNet50, and 2560 for VIRCHOW. None of the data from this study were used to train the models for deep feature extraction, ensuring no data leakage occurred.

Nuclei morphology features

NPC is characterised by significant infiltration of leukocytes into the tumour microenvironment (TME). The abundance and composition of tumour-infiltrating lymphocytes (TILs) have been shown to correlate with patient prognosis in NPC[8]. Additionally, the morphological characteristics of tumour cells have been reported to contribute to the aggressiveness of NPC[9] . For these reasons, we included morphological features representing the size and shape of both tumour and lymphocyte nuclei in the image patches as node features.

Size of nuclei was quantified using measurements such as contour area, convex-hull area, equivalent diameter, minor axis length, major axis length, perimeter, bounding box area, and radius. Furthermore, nuclear shape was quantified using features such as roundness, eccentricity, solidity, and orientation. In total, 12 morphological features were analysed.

These features were extracted from both lymphocyte and tumour nuclei in each image patch. To aggregate the features at the patch level, we calculated the mean, standard deviation, kurtosis, range, and skewness for each nuclear type. This resulted in a

morphological feature vector with a total length of 144 (12 features × 2 nuclei types × 6 statistical measures).

Clinical features

To incorporate clinical information into our multimodal GNN prognostic model, we used 7 clinical features: age, sex, smoking status, family history of cancer, T stage, N stage, and overall stage. We applied one-hot encoding for categorical features and standardisation for numerical features before passing them to the model.

$$Z = \frac{X - \mu}{\sigma}$$

Graph construction

A graph is a data structure consisting of nodes (vertices) and edges (connections). This structure is well-suited for modelling large images, such as whole slide images (WSIs). The WSI graph was constructed by connecting nodes, each represented by image patches. The node features consisted of vectors obtained from the concatenation of deep features and morphological features. Connections between nodes were established using Delaunay triangulation, with a threshold of 2,000 pixels. This threshold was empirically defined to limit connections between nodes within a single tissue area. Furthermore, to capture spatial relationships and heterogeneity in the tumour microenvironment, edges were weighted based on the cosine similarity of node features. Cosine similarity assigns a score between 0 and 1 to the feature vectors, where 1 indicates that the vectors are identical, and 0 indicates they are completely unrelated. The edge weights capture both spatial relationships and heterogeneity in the tumour microenvironment. Several patients in the SYSUCC cohort had multiple slides, and some slides contained multiple tissue areas. To accommodate this complexity, we combined all tissue areas for each patient into one large graph, consisting of disjointed smaller graphs.

GNN model

In a graph of G, the graph is represented by $G = (V, E)$, where V denotes the set of nodes and E represents the set of edges that link these nodes. The graph structure is encoded in adjacency matrix A, where A_{ij} is equal to 1 if node i and j are connected, and A_{ij} is equal to 0 if otherwise. In our WSI graph G, the V was image patches which represented by concatenation of deep features and morphological features of tumour and lymphocytes nuclei. E was constructed using Delaunay triangulation and weighted by a cosine similarity function. This approach captures tissue structure, which is important for representing the tumour microenvironment. In addition, the weighted E enable the model to highlight patterns and relationship in regions while reduce impact of irrelevant regions.

A GNN learns from graph by updating each node's feature representation through the aggregation of information from its neighbour, a process formally known as message passing. Various message passing algorithm have been proposed. In this study, we used Graph Convolution Networks (GCN)[10] proposed by Kipf and Welling for message passing.

In GCN, node representations are updated through a convolution operation defined as follow:

$$H^{(l+1)} = \sigma\left(\tilde{A}H^{(l)}W^{(l)}\right)$$

where $H^{(l)}$ is the feature matrix in the lth layer and $H^{(0)} = X$. In this study, $\sigma(.)$ denotes an activation function, specifically Leaky-ReLU[11], which is more suitable than standard ReLU for complex and diverse structure such as graphs. $W^{(l)}$ is a learnable weight matrix for layer l.

Each successive GCN layers learn new feature matrix $H^{(l)}$ that represents each node along with its neighbourhood information before aggregation operation in pooling layer. We used self-attention graph (SAGPooling)[12] with hierarchical architecture as pooling layer to aggregate node information. SAGPooling computes an attention score s for each node V using linear transformation followed by non-linear activation, specifically $tanh$ in this study.

$$s = tanh\left(H^{(l)} \cdot w\right)$$

The GNN in this study comprised of four layers, each of which includes a GCN and SAGPooling layer. The output of each block is aggregated in the readout step using the operation

$$h^{(j)} = \frac{1}{N}\sum_{i=1}^{N} x_i \,||max_{i=i}^{N} x_i$$

and is summarised by summation operation in final readout layer. This latent representation of graph is then concatenated with latent representation of clinical information. The combined embedding is then passed to the final fully connected layer, which has a single output node.

Loss function

The model was trained using regularised negative log-likelihood (NLL) loss derived from the partial likelihood of the Cox proportional hazard model. The Cox model assumes a hazard function at time t for individual with covariates x, denoted as $h(t|x)$, is proportional to a baseline hazard function $h_0(t)$.

Let $l(\theta)$ denote the NLL loss:

$$l(\theta) \coloneqq -\frac{1}{N_{E=1}} \sum_{i:E_i=1} \left(\widehat{h_\theta}(x_i) - \log \sum_{j \in R(T_i)} e^{\widehat{h_\theta}(x_j)} \right) + \lambda \cdot |\theta|_2^2$$

where N_E is the number of observed (uncensored) events ($E = 1$). Here, $\widehat{h_\theta}(x_i)$ is the predicted risk score of individuals i under the model parameters θ, as represented by features vector x_i. The risk set $R(T_i)$ consists of all individuals j who are still at risk at time T_i, including those whose event or censoring time satisfies $T_j \geq T_i$.

The objective is to minimise the negative log-partial likelihood under the assumption that higher risk scores correspond to worse outcomes (i.e., shorter time to the event). For each observed event ($E_i = 1$), the risk score $\widehat{h_\theta}(x_i)$ is compared to the normalised cumulative risk of individuals in the risk set $R(T_i)$. Finally, the loss is regularised using L2 regularisation with strength λ, which penalises large parameters to reduce overfitting and improve generalisation.

Multimodal GNN for NPC survival (GNPC)

GNPC is a multimodal model comprising two concurrent networks: a graph neural network (GNN) and a fully connected network (FCN). The latent embeddings from each network were concatenated into an intermediate network before being passed into the final fully connected network.

For the GNN, we used a graph convolutional network (GCN) as the convolutional layer, followed by a Leaky-ReLU non-linear activation layer. All FCN layers utilised the scaled exponential linear unit (SELU) as the activation function. To mitigate overfitting, dropout with a rate of 0.4 was applied to each layer of the FCN. Additionally, 1D batch normalisation was employed in the FCN to stabilise training.

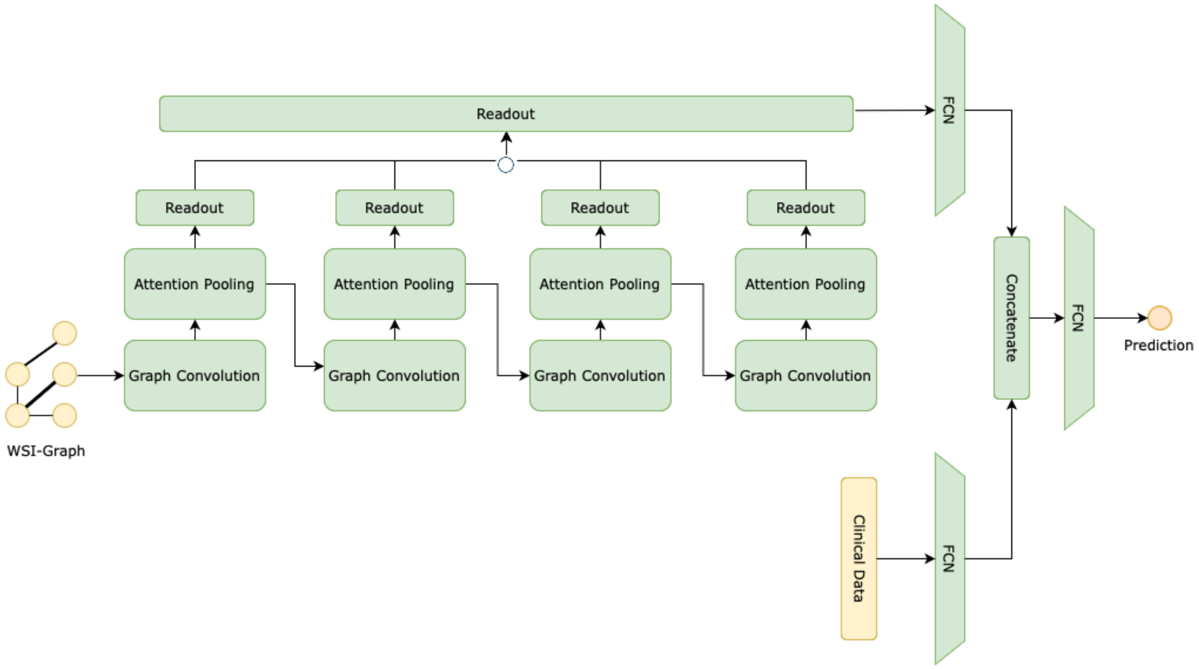

Figure S2 GNPC architecture

We used stochastic gradient descent (SGD) with learning rate of 1e-3 to train GNPC. A batch size of 64 and a maximum epoch of 100 were employed during training. Additionally, to prevent overfitting and improve generalisation, L2 regularisation with a coefficient of 1e-4, weight decay of 1e-5 and early stopping were applied.

Foundation models for survival prediction

We conducted comparative studies to assess the performance of the state-of-the-art foundation models (VIRCHOW [4], CONCH [5], UNI [6] and DINO [7]) for survival prediction of NPC. Additionally, we used deep features from widely used deep learning model ResNet50[13] as a baseline. All models were trained using 5-fold cross-validation derived from the discovery set. To evaluate model performance, we measured the C-index and the p-value from the log-rank test of the predicted risk scores. Risk scores in the validation set were stratified into low- and high-risk groups based on the median risk score value from the training set. We aggregated the results of 5-fold cross-validation by calculating the mean and standard deviation of the C-index and the median p-value from the log-rank test which are showed in the figure below.

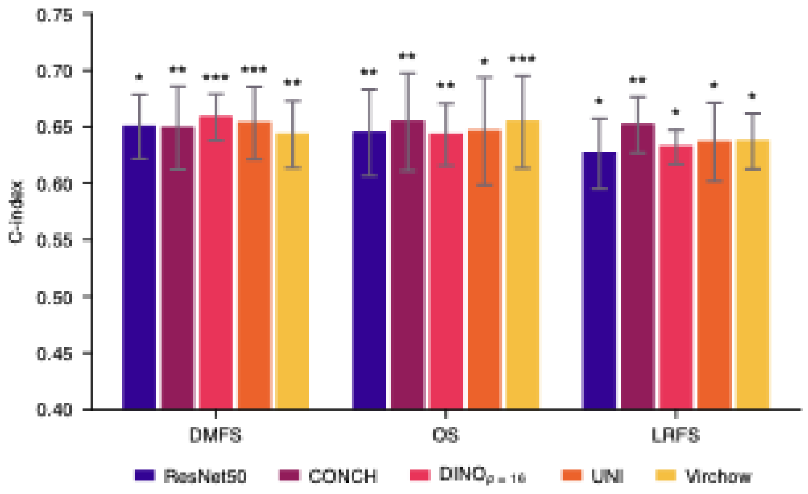

Figure S3 Comparative study on foundation model

Based on the median p-values across folds, all models in the experiments demonstrated statistically significant results. However, no model was particularly prominent, as all models show comparable predictive accuracy across the evaluated survival endpoints, with performance differences being relatively minor. In DMFS and OS, some foundation models deliver slightly worse performance compared to the baseline model, ResNet50, which was pretrained on non-histology images. To determine the deep features for the final model, we performed a top 3 voting based on the mean C-index across survival endpoints, resulting in the selection of CONCH 30 for deep feature extractor.

Model selection

To ensure the generalisability and robustness of our model, we trained it on the SYSUCC cohort and evaluated its performance on unseen data from both the SYSUCC (internal test set) and CUHK (external test set) cohorts. We split the larger SYSUCC cohort into a discovery set (80%) and an internal testing set (20%), stratified by survival time across each survival endpoint. The entire CUHK cohort was reserved for external testing, providing an independent dataset to validate the model's generalisability.

To optimise model hyperparameters, we conducted 5-fold cross-validation on the discovery set. Models from the cross-validation study were evaluated using the C-index and the log-rank test of the Kaplan-Meier (KM) curve. For KM curve evaluation, we used the median risk score from the training set to stratify patients in the test set into low- and high-risk groups. We reported the mean and standard deviation of the C-index, as well as the median p-value from the log-rank test.

For final model evaluation, we trained on the full discovery set for each survival endpoint and then tested the model on both the internal and external test sets. In both the internal

and external test sets, we applied the median risk score from the discovery set to define risk groups. A p-value of <0.05 was used as the threshold for statistical significance in all tests.

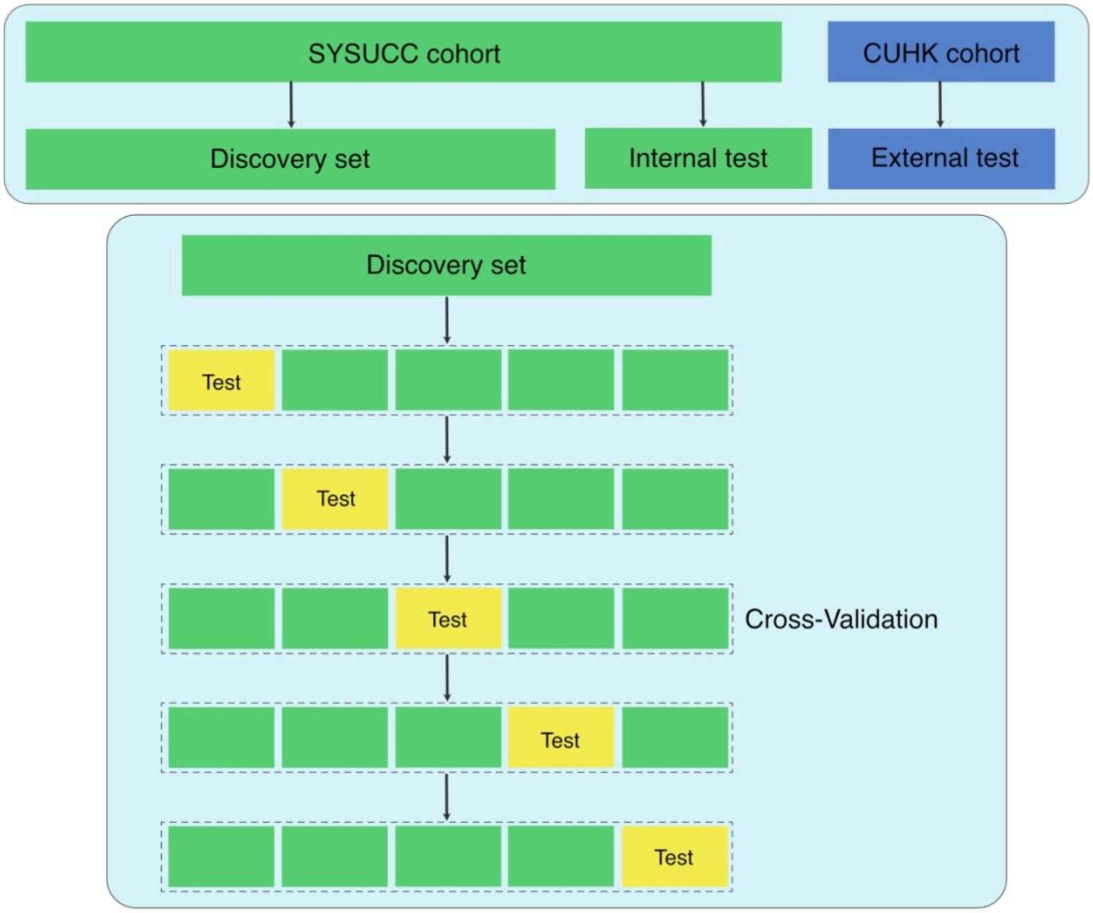

Figure S4 Model selection

Statistical analyses

Risk groups were compared using the log-rank test in Kaplan-Meier (KM) curve analysis. The concordance index (C-index) was employed to assess the predictive performance of the model.

Differences between groups were tested using Student's t-test for continuous variables and Fisher's exact test for categorical variables. Correlation analysis was performed using the Pearson correlation coefficient. Cox proportional hazards (Cox-PH) models were used to evaluate the prognostic value of the GNPC risk score and established risk factors in both univariate and multivariate analyses. A p-value < 0.05 was considered statistically significant, with significance levels denoted as $p < 0.05$ (*), $p < 0.01$ (**), and $p < 0.001$

(***). The main pipeline and downstream analyses were implemented in Python version 3.10. Statistical analyses were conducted using the lifelines package (https://lifelines.readthedocs.io/en/latest/) and the scikit-survival package (https://scikit-survival.readthedocs.io/en/stable/).

Salient region analyses

The pooling layer is a crucial component in GNNs, as it creates a refined representation of the graph by reducing and selecting the most relevant nodes for the prediction task. In our model, we utilised SAG pooling [12], which enables the model to focus on specific nodes based on their relevance to the prediction task. This attention mechanism provides a way to interpret the model, allowing us to identify which parts of the tissues or slides are important for the prediction results and examine the TME in these salient regions

We selected the top 10% most relevant patches based on node's attention score. Nuclei features in these relevant patches were then aggregated by calculating the mean at the slide level and subsequently at the patient level. Statistical differences between groups were assessed by comparing the mean of these aggregated results. From this salient region, we analysed the immune profile across GNPC risk groups by measuring the density of the inflammatory cells, as well as the entropy and ratio of tumour (neoplasm) cells to inflammatory cells. Density was defined as the abundance of inflammatory cells per mm^2, while entropy and ratio described the heterogeneity of tumour and inflammatory cells. Together, these metrics reflect the immune activity. Additionally, we investigated tumour morphology, focusing on shape ellipticity**.** Ellipticity was quantified using eccentricity, where a higher value indicates greater deviation from a perfect circular shape.

Correlation between GNPC risk score with EBV-DNA levels and LMP1 status

We examined correlation between EBV DNA levels and the GNPC risk score in internal validation cohort. First, we stratified patients into low and high-EBV DNA groups using threshold of 4,000 [14,15] and compared the distribution of these groups within low and high-risk group of GNPC. Secondly, we measured correlation between GNPC score and EBV DNA levels.

We investigate the association between GNPC scores and LMP1 expression in an external validation cohort respectively (Fig S5). LMP1 expression was determined by immunohistochemical staining, assessed using a proportion score and an intensity score [16]. Patients were stratified into absence/low and high LMP1 expression groups based on a H-score threshold of 100 [1], and we compared its distribution across GNPC risk groups.

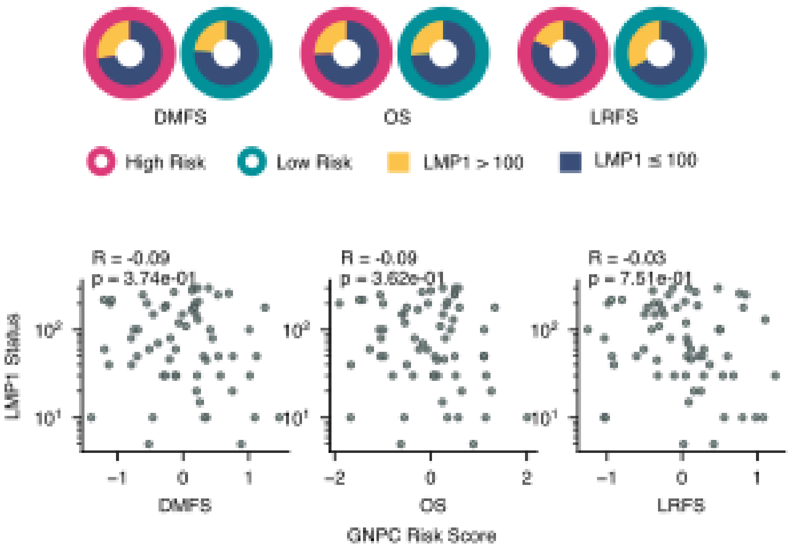

Figure S5 GNPC score association with LMP1 status

Association between immune characteristics and gene mutation in NPC

We compared how lymphocyte density differs between groups of patients with non-mutated and mutated genes (Figure 6). Inflammatory density was measured by calculating the number of inflammatory cells nuclei in salient tumour regions. This analysis was conducted on an external validation (CUHK) cohort.

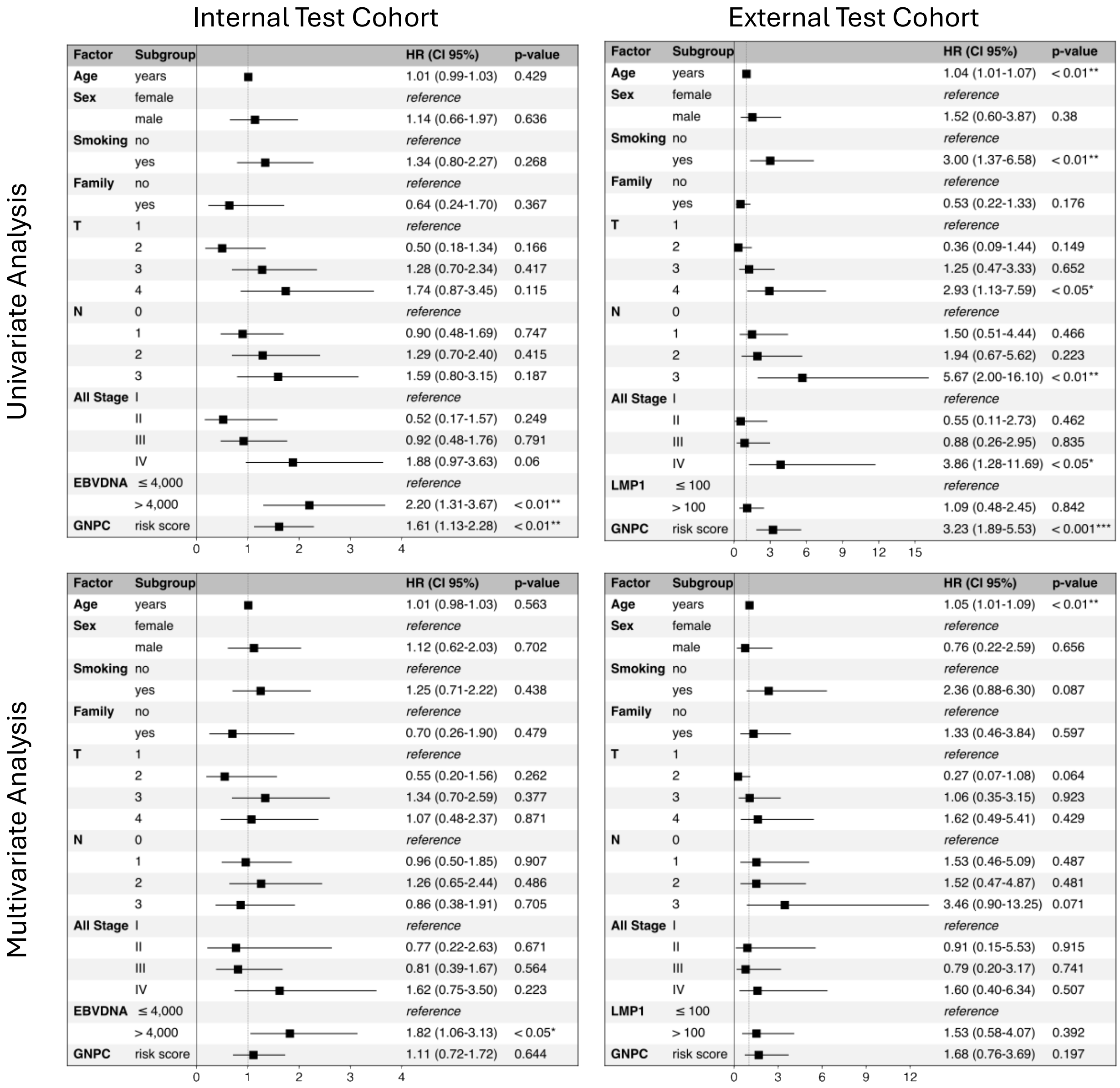

Figure S6 Cox-PH analysis on overall survival cases

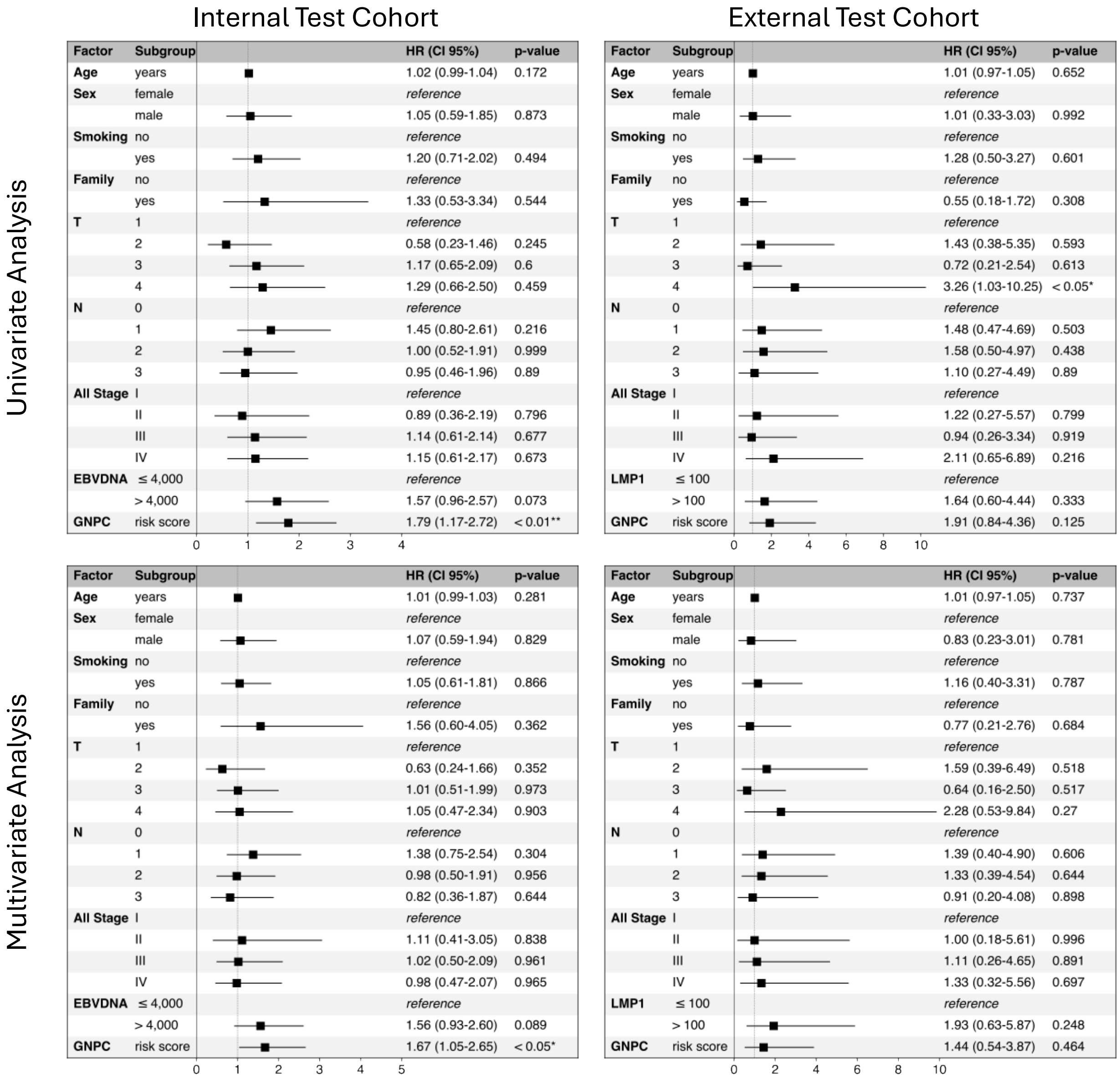

Figure S7 Cox-PH analysis on local recurrence cases

The prognostic performance of the GNPC model was compared with that of the TNM stage for three survival endpoints: DMFS, OS, and LRFS. For each covariate, the concordance index (C-index) was computed, and the highest value within each cohort and survival endpoint is denoted in yellow.

Table S2. Performance comparison between GNPC and TNM staging in the internal validation cohort for the DMFS endpoint

Covariate	Cohort	Endpoint	C-Index
T	Internal Validation	DMFS	0.597
N	Internal Validation	DMFS	0.619
GNPC	Internal Validation	DMFS	0.683
All Stage	Internal Validation	DMFS	0.631

Table S3. Performance comparison between GNPC and TNM staging in the external validation cohort for the DMFS endpoint

Covariate	Cohort	Endpoint	C-Index
All Stage	External Validation	DMFS	0.716
GNPC	External Validation	DMFS	0.775
N	External Validation	DMFS	0.743
T	External Validation	DMFS	0.685

Table S4. Performance comparison between GNPC and TNM staging in the internal validation cohort for the LRFS endpoint

Covariate	Cohort	Endpoint	C-Index
All Stage	Internal Validation	LRFS	0.537
GNPC	Internal Validation	LRFS	0.626
N	Internal Validation	LRFS	0.485
T	Internal Validation	LRFS	0.563

Table S5. Performance comparison between GNPC and TNM staging in the external validation cohort for the LRFS endpoint

Covariate	Cohort	Endpoint	C-Index
All Stage	External Validation	LRFS	0.627
GNPC	External Validation	LRFS	0.647
N	External Validation	LRFS	0.532
T	External Validation	LRFS	0.644

Table S6. Performance comparison between GNPC and TNM staging in the internal validation cohort for the OS endpoint

Covariate	Cohort	Endpoint	C-Index
All Stage	Internal Validation	OS	0.640
GNPC	Internal Validation	OS	0.657
N	Internal Validation	OS	0.592
T	Internal Validation	OS	0.597

Table S7. Performance comparison between GNPC and TNM staging in the external validation cohort for the OS endpoint

Covariate	Cohort	Endpoint	C-Index
All Stage	External Validation	OS	0.759
GNPC	External Validation	OS	0.814
N	External Validation	OS	0.740
T	External Validation	OS	0.694

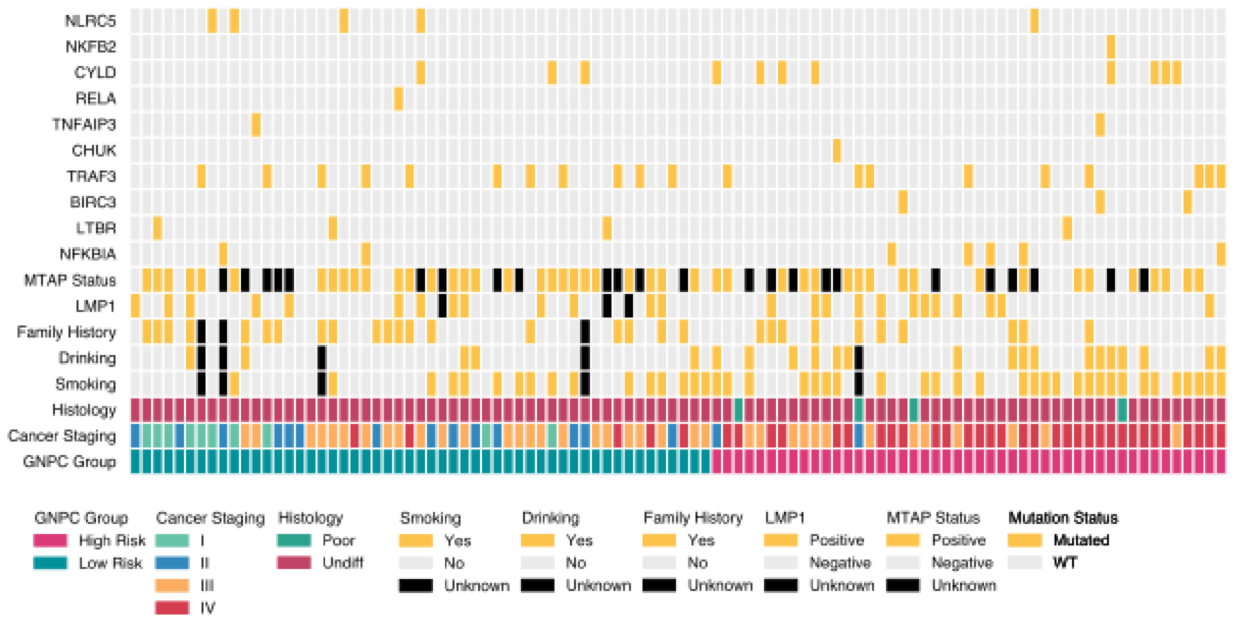

Figure S8 Heatmap risks factor in overall survival cases

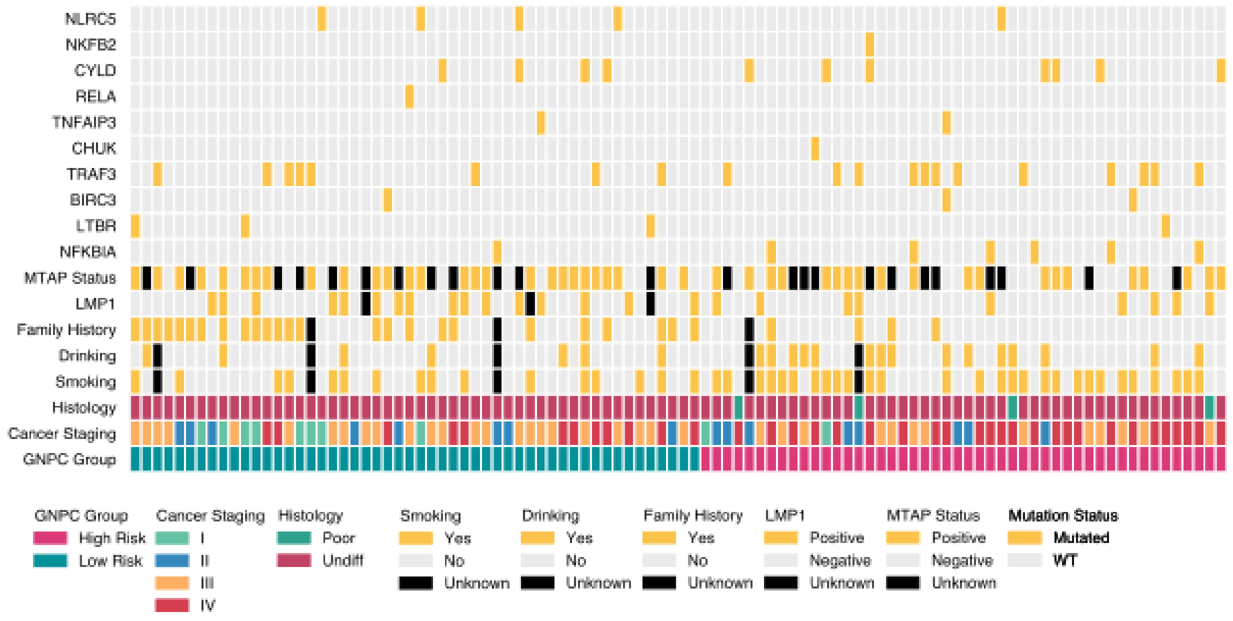

Figure S9 Heatmap risks factor in local recurrence cases

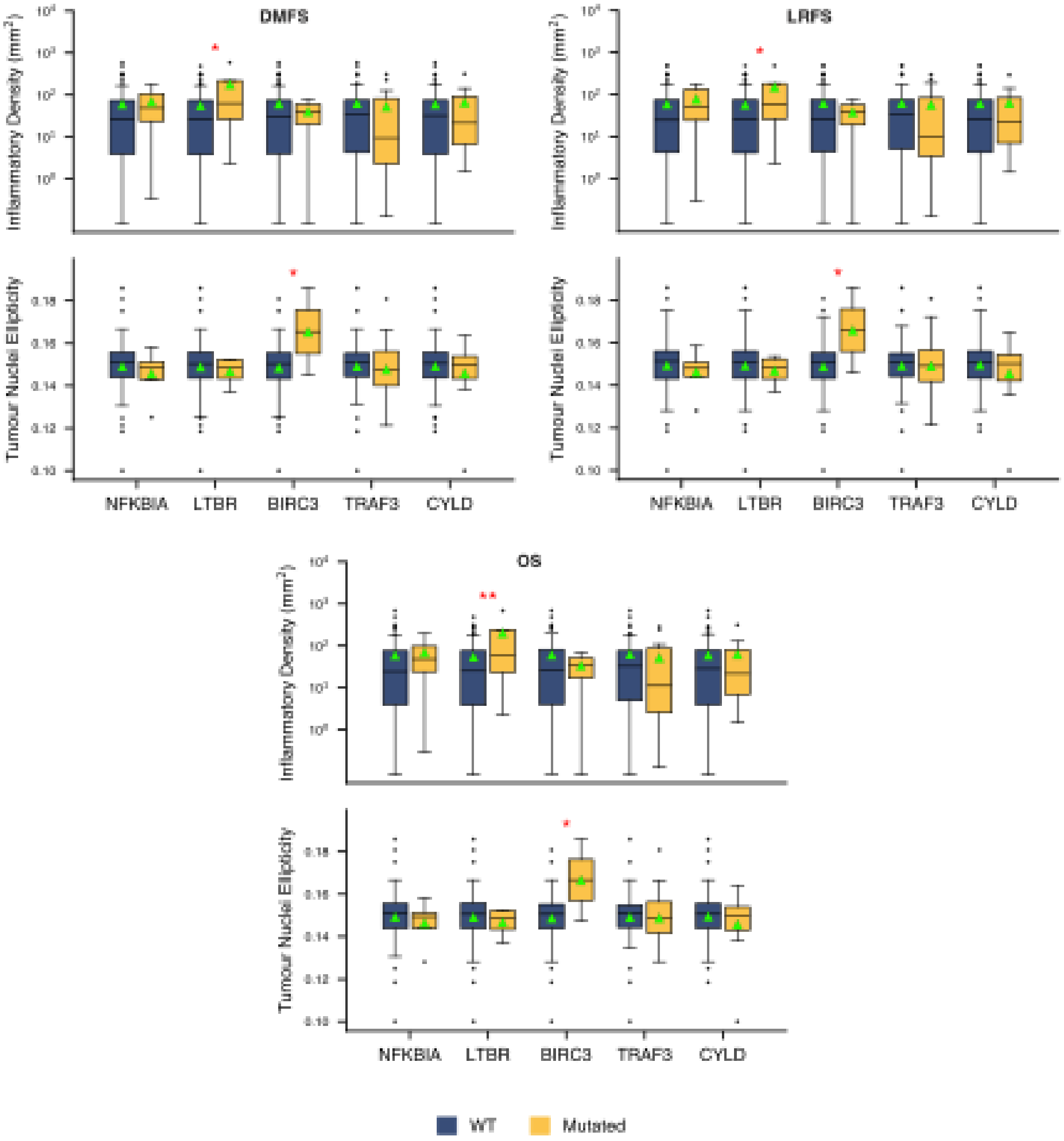

Figure S10 Association between gene alteration and immune cell density and tumour nuclei ellipticity/spindle tumour cells across all survival endpoints. Statistical significance is denoted by a red star. Significance levels denoted as (*) ($p < 0.05$), ** ($p < 0.01$), and *** ($p < 0.001$).

References

1. Li YY, Chung GTY, Lui VWY, et al. Exome and genome sequencing of nasopharynx cancer identifies NF-κB pathway activating mutations. *Nat Commun*. 2017;8(1):14121. doi:10.1038/ncomms14121

2. Bruce JP, To KF, Lui VWY, et al. Whole-genome profiling of nasopharyngeal carcinoma reveals viral-host co-operation in inflammatory NF-κB activation and immune escape. *Nat Commun*. 2021;12(1):4193. doi:10.1038/s41467-021-24348-6

3. Janowczyk A, Zuo R, Gilmore H, Feldman M, Madabhushi A. HistoQC: An Open-Source Quality Control Tool for Digital Pathology Slides. *JCO Clin Cancer Inform*. 2019;(3):1-7. doi:10.1200/CCI.18.00157

4. Vorontsov E, Bozkurt A, Casson A, et al. A foundation model for clinical-grade computational pathology and rare cancers detection. *Nat Med*. 2024;30(10):2924-2935. doi:10.1038/s41591-024-03141-0

5. Lu MY, Chen B, Williamson DFK, et al. A visual-language foundation model for computational pathology. *Nat Med*. 2024;30(3):863-874. doi:10.1038/s41591-024-02856-4

6. Chen RJ, Ding T, Lu MY, et al. Towards a general-purpose foundation model for computational pathology. *Nat Med*. 2024;30(3):850-862. doi:10.1038/s41591-024-02857-3

7. Kang M, Song H, Park S, Yoo D, Pereira S. Benchmarking Self-Supervised Learning on Diverse Pathology Datasets.

8. Liu W, Chen G, Zhang C, et al. Prognostic significance of tumor-infiltrating lymphocytes and macrophages in nasopharyngeal carcinoma: a systematic review and meta-analysis. *Eur Arch Otorhinolaryngol*. 2022;279(1):25-35. doi:10.1007/s00405-021-06879-2

9. Luo W, Yao K. Molecular Characterization and Clinical Implications of Spindle Cells in Nasopharyngeal Carcinoma: A Novel Molecule-Morphology Model of Tumor Progression Proposed. Busson P, ed. *PLoS ONE*. 2013;8(12):e83135. doi:10.1371/journal.pone.0083135

10. Kipf TN, Welling M. Semi-Supervised Classification with Graph Convolutional Networks. Published online February 22, 2017. Accessed November 10, 2023. http://arxiv.org/abs/1609.02907

11. Maas AL, Hannun AY, Ng AY. Rectifier Nonlinearities Improve Neural Network Acoustic Models. *Proc Int Conf Mach Learn*. Published online 2013.

12. Lee J, Lee I, Kang J. Self-Attention Graph Pooling. In: *Proceedings of the 36th International Conference on Machine Learning*. PMLR; 2019:3734-3743. Accessed November 5, 2024. https://proceedings.mlr.press/v97/lee19c.html

13. He K, Zhang X, Ren S, Sun J. Deep Residual Learning for Image Recognition. In: *2016 IEEE Conference on Computer Vision and Pattern Recognition (CVPR)*. IEEE; 2016:770-778. doi:10.1109/CVPR.2016.90

14. Wibawa MS, Zhou JY, Wang R, et al. AI-Based Risk Score from Tumour-Infiltrating Lymphocyte Predicts Locoregional-Free Survival in Nasopharyngeal Carcinoma. *Cancers*. Published online 2023.

15. Tang LL, Guo R, Zhang N, et al. Effect of Radiotherapy Alone vs Radiotherapy With Concurrent Chemoradiotherapy on Survival Without Disease Relapse in Patients With Low-risk Nasopharyngeal Carcinoma: A Randomized Clinical Trial. *JAMA*. 2022;328(8):728. doi:10.1001/jama.2022.13997

16. Kang W, Tong JHM, Chan AWH, et al. Yes-Associated Protein 1 Exhibits Oncogenic Property in Gastric Cancer and Its Nuclear Accumulation Associates with Poor Prognosis. *Clin Cancer Res*. 2011;17(8):2130-2139. doi:10.1158/1078-0432.CCR-10-2467
